# ACO:lossless quality score compression based on adaptive coding order

**DOI:** 10.1186/s12859-022-04712-z

**Published:** 2022-06-07

**Authors:** Yi Niu, Mingming Ma, Fu Li, Xianming Liu, Guangming Shi

**Affiliations:** 1 School of artificial intelligence, Xidian University, Xian, 710071 China; 2 The Pengcheng Lab, Shenzhen, 518055 China

**Keywords:** High-throughput sequencing, Quality score compression, Lossless compression, Adaptive coding order

## Abstract

**Background:**

With the rapid development of high-throughput sequencing technology, the cost of whole genome sequencing drops rapidly, which leads to an exponential growth of genome data. How to efficiently compress the DNA data generated by large-scale genome projects has become an important factor restricting the further development of the DNA sequencing industry. Although the compression of DNA bases has achieved significant improvement in recent years, the compression of quality score is still challenging.

**Results:**

In this paper, by reinvestigating the inherent correlations between the quality score and the sequencing process, we propose a novel lossless quality score compressor based on adaptive coding order (ACO). The main objective of ACO is to traverse the quality score adaptively in the most correlative trajectory according to the sequencing process. By cooperating with the adaptive arithmetic coding and an improved in-context strategy, ACO achieves the state-of-the-art quality score compression performances with moderate complexity for the next-generation sequencing (NGS) data.

**Conclusions:**

The competence enables ACO to serve as a candidate tool for quality score compression, ACO has been employed by AVS(Audio Video coding Standard Workgroup of China) and is freely available at https://github.com/Yoniming/ACO.

## Background

Sequencing technology has gradually become a basic technology widely used in biological research [1]. Obtaining genetic information of different organisms can help us to improve our understanding of the organic world. In the past decades, the price of human whole genome sequencing (WGS) has dropped to less than $1000, with a faster declining speed over the Moore’s Law expected [2]. In this case, the number of next-generation sequencing (NGS) data grows exponentially, even exceeds that of astronomical data [3]. How to efficiently compress the DNA data generated by large-scale genome projects has become an important factor restricting the further development of the DNA sequencing industry. Therefore, it is necessary to compress NSG data for more convenient storage and transmission.

To be specific, there are two major problem in the compression of DNA data: the nucleotide compression and quality score compression. The quality values takes more than half of the compression data and has been shown to be more difficult to compress than the nucleotide data [4, 5]. To improve the compression ratio of the total file size, it is necessary to make separate efficiency improvements for the quality fraction. With the development of assembling techniques [6], the nucleotide compression have achieved significant improvement which makes the quality score compression problem to be one of the main bottle-necks in the current DNA data storage and transfer applications.

The quality score (QS) represents the confidence level of every base characters in the sequencing procedure for the next-generation sequencing (NGS) data, but with a much larger alphabets (41–46 distinct levels). There are now many more instrument manufacturers and that they predominantly still have a large number of discrete quality values. Studying the data characteristics of quality scores is very helpful for compression, [7] reveals that there are strong correlations among adjacent quality score, which can be regarded as the foundation of the current lossless quality score compression pipeline: (1) using Markov model to estimate the conditional probability of the quality score; (2) traversing every position of the reads via a raster scan order; (3) encoding the quality score via arithmetic or range coding.

Although many methods have been proposed for lossy compression of quality scores [8–11], it is particularly important to preserve the original data. Therefore, we focus on lossless compression, and there are many recent neural network-based compression methods that combine recurrent neural network predictors with arithmetic encoders to losslessly compress genomic datasets [12, 13]. However, the genetic data generated by different sequencing machines have different distributions, which makes network training-based methods require separate training for each data and is not conducive to broad application. Based on the above pipeline, three distinguished lossless compressor have been proposed GTZ [14], Quip [15] and FQZcomp [5]. The only differences among these three works are the Markov model orders and context quantization strategies, thus the compression ratio varies around $0.1\%$ [16, 17], depending on the data distribution. A negative view is unavoidably raised that there is not much rooms for the further improvement of lossless compression ratio.

In this paper, by reinvestigate the sequencing process, we reveal two main drawback of the existing raster scan based quality score compression strategy. Firstly, the raster scan order is a “depth-first” traverse strategy of the read. However, as it indicated in [7], the quality score has a descent trend along one single read. This makes the piece-wise stationary assumption of Markov modeling untenable. Secondly, considering that the sequencing process is conduced by 2D multi-spectral imaging [18], but the FASTQ file simply stores the quality score into a stack of 1D signals. The raster-scan based techniques compress every reads independently which fails to explore the potential 2D correlations the spatial-adjacent reads (not the adjacent reads from FASTQ files).

To overcome the above two drawbacks, we propose a novel quality score compression technique based on adaptive coding order(ACO) for the next-generation sequencing (NGS) data. Different from general compression methods, ACO is a special compressor for quality scores, so it considers the distribution characteristics of more quality score data. In general, ACO has three contributions: (1) according to the internal correlation of quality scores, the row mean is introduced as the context information; (2) the base information is introduced as the context information according to the sequencing principle and quantifies the composite context model; (3) adopt serpentine coding sequence. The main objective of ACO is to traverse the quality score along the most relative directions, which can be regarded as a reorganization of the stack of independent 1D quality score vectors into highly related 2D matrices. Another improvement of the proposed ACO technique over the existing techniques is the compound context modeling strategy. As we will explain the details in Section of Method, instead of the adjacent QS values, the ACO context models consists of two additional aspects: (1) the global average of every reads; (2) the variant of DNA bases. The compound context model not only benefits the probability estimation and arithmetic coding, more importantly, in the implementation, it prevents ACO from multiple random access of the input FASTQ file: the compressing process can be accomplished in only one-path, at the cost of some context dilution and side-information.

Experimental results show that the proposed ACO technique achieves the state-of-the-arts performances for the lossless quality score compression, which achieves more than $5\%$ gains in the compression ratio over FQZcomp [5]. The only drawback of ACO is the memory cost, comparing with FQZcomp, ACO requires 400*M* and 4*G* additional memory to buffer the 2D quality score matrixes and store the compound context models respectively, which should no longer been a big problem for the current PC.

## Insight

In this section, we will first analyze the data characteristics of the quality score, and illustrate that the coding sequence will have a certain impact on the quality score compression through specific examples, so as to promote us to compress the quality score along the direction with the strongest data correlation. Secondly, by analyzing the sequencing principle and the generation process of FASTQ file, we explore the extra relevance in the quality score data to build a novel composite context quantification model. The results of these analyses constitute the generation of our innovations, resulting in the lightweight and portable quality score compressor ACO.

### Impact of coding order

**Fig. 1:**
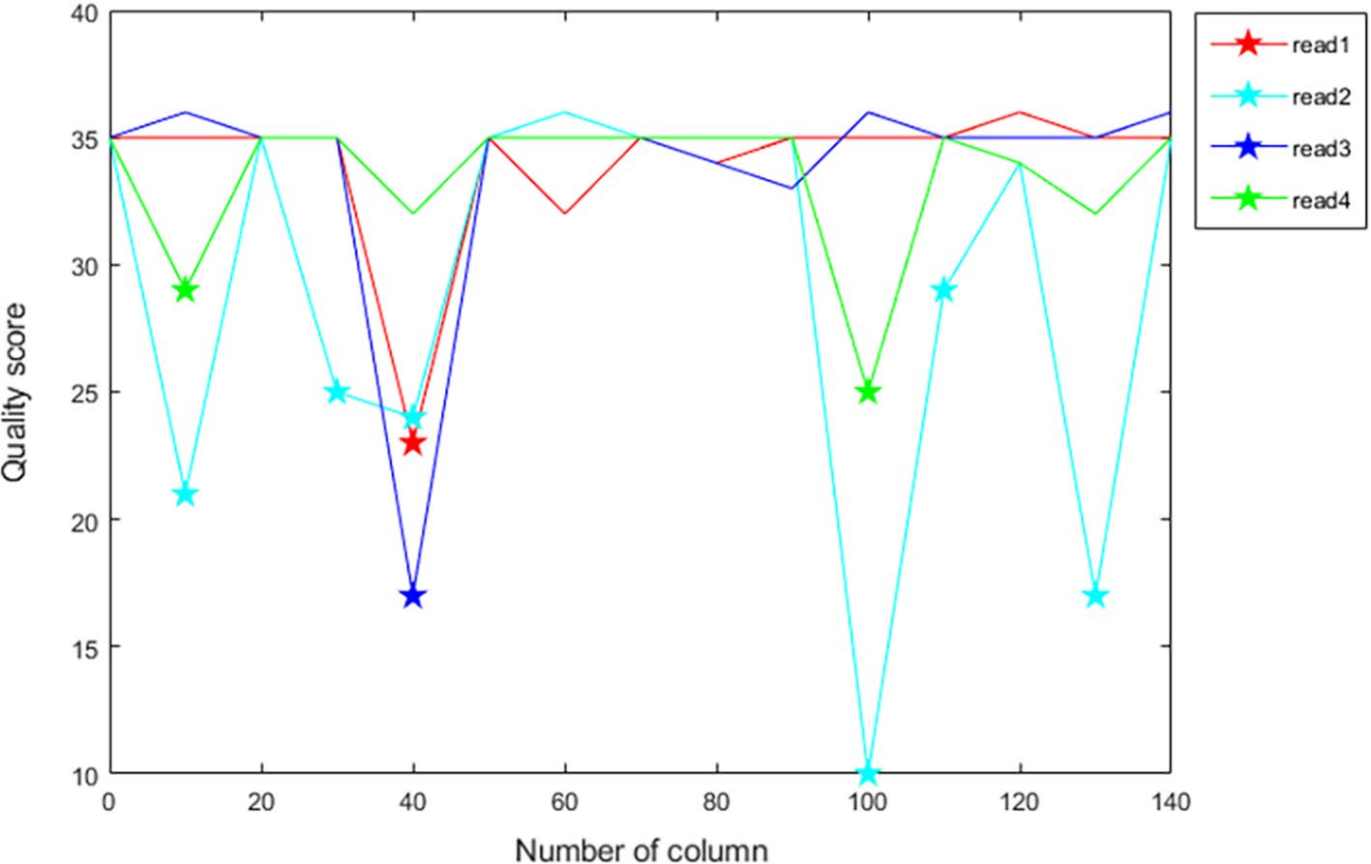
Quality score distribution curve of *ERR*2438054

The quality score represent the estimation of the probability of the corresponding nucleotide error in the reads, and it is the evaluation of the reliability of the base character. This information is used for both the quality control of the original data and the downstream analysis. We give the distribution of the quality score of the four reads of ERR2438054 in Fig.1, it can be seen that due to the influence of noise (points marked by a five-pointed star), the quality score is a random and unstable signal, and there is a strong correlation between adjacent quality score. Therefore, we can use these characteristics of quality score to change the coding order to improve the compression ratio. Changing the order doesn’t sound like changing the entropy value, because according to the information theory, the information quantity of the source is a function of probability, which is represented by the information entropy of the source. However, since the adaptive arithmetic encoder is used in coding, the encoder will update the symbol probability regularly, so changing the order can reduce the size of the bitstream. The discussion on the principle of arithmetic encoder is not the main content of this paper, so we just give a test experiment to show the influence of coding order on compression results. Firstly, we create two random signals *X* and *Y*, assuming that the random signals *X* and *Y* are two different Gaussian signals, and let $Z1 = X + Y$, *Z*1 means that *X* and *Y* are connected in series, this does not mean the add up corresponding values. Then, randomly disturb the distribution of *Z*1 and record it as *Z*2, compared with *Z*1 with two Gaussian distributions, the *Z*2 distribution after shuffled is more stable, then sort the distribution of *Z*1 by size and record it as *Z*3. Finally, three groups of different signals are encoded by 0-order arithmetic encoder, the result of the bitstream is $S(Z3)<S(Z2)<S(Z1)$ ($S(\cdot )$ represents the size of the entropy). This is because the sorting process is equivalent to placing the data together with similar distribution and strong correlation. The coding after changing the order can better cooperate with the probability update mechanism of adaptive arithmetic encoder.

**Fig. 2:**
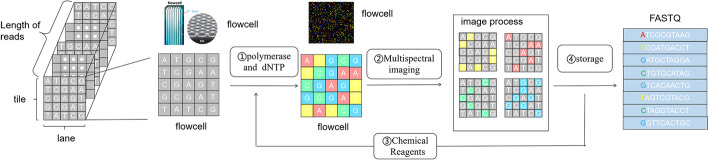
schematic diagram of sequencing principle

### Mining more relevant information

Take the current widely used Hiseq sequencing platform as an example, the sequencing process consists of three steps: (1) construction of DNA library, (2) generating DNA cluster by bridge PCR amplification and (3) sequencing. In this paper we study the sequencing step to mining more inherent correlations among the quality score to aid the compression task. The basic principle of sequencing is based on multi-spectral imaging of the flowcell.

As shown in in Fig.2, the sequencing process consists of five steps. In step 1, the polymerase and one type of dNTP are added into the flowcell to activate the fluorescent of the specific clusters. In step 2, the multi-spectral camera takes one shot of the flow cell with the specific wavelength according to the added dNTP. Then in step 3, chemical reagents are adopted to wash out the flowcell to prepare for the next imaging. The above three steps are repeated four times with different dNTPs and different imaging wavelength to get a four channel multi-spectral image. In step 4, based on the captured four channel image, the sequencing machine not only estimate the most likely type of every cluster but also evaluate the confident level of the estimation, which are stored as the bases and quality score respectively. Step1-4 is regarded one sequencing cycle which sequence one position (depth) of all the reads in the flowcell. Thus in step 5, the sequencing cycle is repeated several times and the repeated number of cycles corresponds to the length of the reads.

As we discussed in details as follows, there are three aspects which corresponds to the quality score values: (1) number of cycles, (2) base change and (3) local position of chip.

**Fig. 3:**
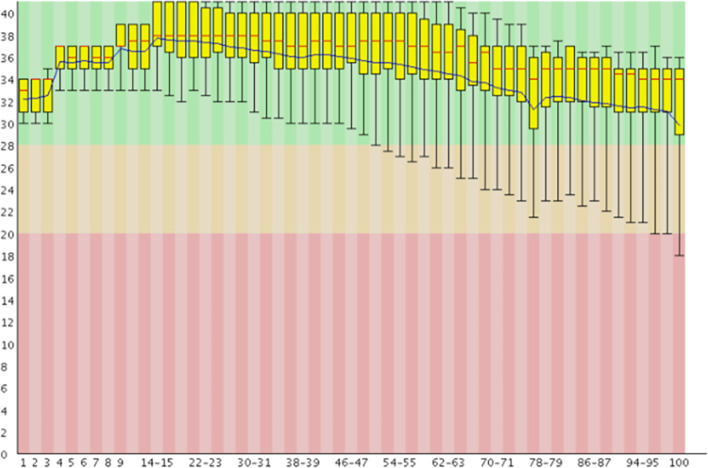
Distribution of quality score made by FASTQ

The number of cycles affects the distribution of quality score. DNA polymerases are used in the process of synthesis and sequencing, at the beginning of sequencing, the synthesis reaction was not very stable, but the quality of the enzyme was very good, so it would fluctuate in the high-quality region. With the progress of sequencing, the reaction tends to be stable, but the enzyme activity and specificity gradually decreases, thus the cumulative error is gradually amplified. As a result, the probability of error increases, and the overall quality score shows a downward trend. As shown in Fig.3(generated by FastQC [19]), with the progress of sequencing, the mean value of quality score decreases gradually, while the variance is increasing. Therefore, it is improper to assume every read as a stationary random signal along the traditional raster scan order.

The base change also affects the distribution of quality score. As we discussed before, the recognition of base types in a flowcell is conducted in a four step loop according to the order of dNTP and wavelength. For example, let’s assume the loop order is ‘A-C-G-T’, if the bases of a read is ‘$\dots$AA$\dots$’, after the imaging of the first ‘A’, the flowcell is washed four times until the imaging of the second ‘A’. But if the bases is ‘$\dots$TA$\dots$’, the machine only wash the flowcell once before the imaging of ‘T’. In this way, if the flowcell contains some residuals in the cluster, the former ‘A’ base will affects the imaging process of the latter ‘T’ base, which may cause ambiguity of ‘T’ thus the quality score of ‘T’ drops significantly. Although some machines adopt compound dNTP to replace the four step loop, the residual is still the case that affect the quality score. Therefore, for the quality score compression, the base change should be considered as a side-information to model the marginal probability of every quality score.

The local position of chip affects the distribution of quality score. The flowcell can be regarded as a 2D array that every cluster corresponds to an entry of the array. If the fluorescent of an high amplitude one entry may diffused to the adjacent entries of the array, which is the well-known “cross-talk” phenomena [20]. In other words, there is 2D spatial correlations among the adjacent quality score. However, the stored FASTQ file is a 1D stack of all the reads which ignores the 2d correlation. Therefore, the compression of quality score should mining the potential 2D spatial correlations among the reads.

## Methods

In this section, we will discuss the proposed adaptive coding order (ACO) based quality score compression technique. The two contributions of ACO is (1) using an adaptive scan order to replace the traditional raster scan order which forms a more stationary signal. (2) using a compound context modeling which considers the influence of base change while exploring the potential 2D correlations among quality score.

### Traverse the quality score along the most relative directions

As can be seen from the Fig.3, with the increase of reads length, the column mean decreases but the variance becomes larger, this proves that there is a strong correlation between columns. At the same time, the reduction in the column mean is also consistent with the actual process of specific sequencing, which the quality score has a descent trend along one single read. It has been verified that changing the scan order can improve the performance of the adaptive arithmetic encoder, and coding along the more stable signal will get better coding effect.

**Fig. 4:**
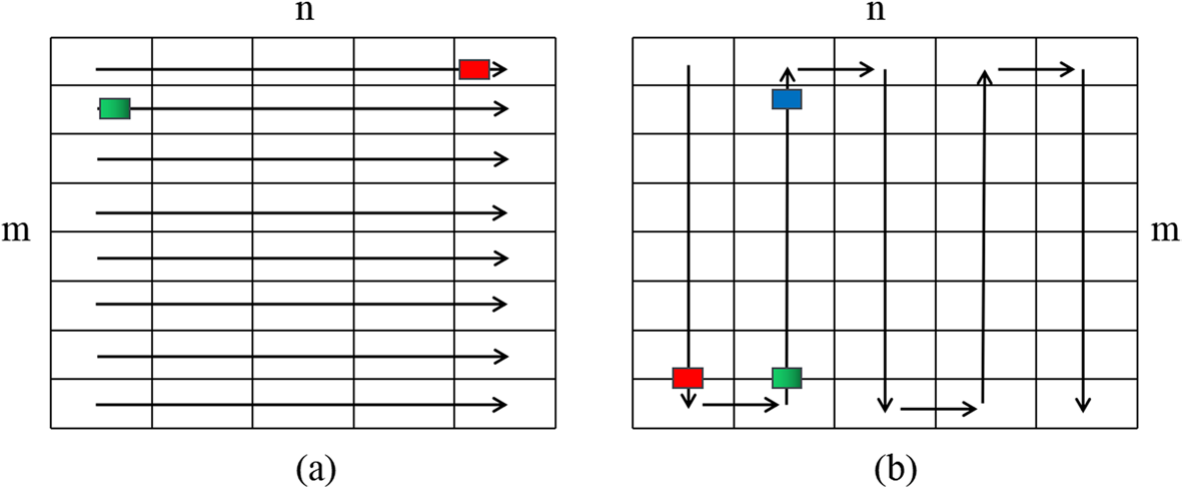
Comparison of traditional scanning and ACO scanning: **a** traditional traversal method; **b** adaptive scan order

All compression methods which based on arithmetic encoder use the scan method in Fig.4a when traversing data encoding. Under this scan method, quality score is encoded line by line, after scan a line, the next step is starting from the beginning of the second line. Obviously, after encoding the last character of the front line, connecting the first character of the next line will cause a great jump and this jump will make the conversion between signals unstable. So we use an adaptive scan order to replace the traditional raster scan order so that realize the stable traversal of the signal which as shown in Fig.4b. The starting point starts with the first element and traverses down the column until the end of a column, then traverses backward up from the end of the next column. Different from the traditional scanning method, ACO adopts a scanning way like the shape of the snake. The reason to use snake traversal is to make the transition between columns more smooth, the end symbols of one column are more relevant to the end symbols of next column, the correlation between red and green symbol is obviously stronger than the correlation between red and blue symbol. Therefore, after the red symbol is encoded, it is more appropriate to select the green symbol than the blue symbol to encode from the second column. By changing the scanning order, the encoding is carried out in a more stable direction. The probability update mechanism of adaptive arithmetic encoder is fully utilized without introducing other factors.

### Compound context modeling

As Section of Declaration explains, the compression of quality score should mining the potential 2D spatial correlations among the reads, so we using a compound context modeling to express the extra relevance in the quality score data. There are two additional aspects are contained in the ACO context model and the first aspect is to get the global average of each read. According to the example in Declaration, it can be seen that adjusting the data order to make the similar symbols cluster together will get good results in compression. As shown in Fig.1, the distribution curves of the four reads are very similar, only some singular points show the differences. So it is an improved strategy to cluster and code the data with similar row distribution, but it will take a lot of steps to calculate the distribution of each row, and cluster similar rows will also bring the loss of time and space. We calculate the mean information of each row to reflect its distribution, and classify the rows with the same mean value. For the row information, the mean is a measure standard of stationarity, rows with the same mean value can be approximately regarded as basically the same distribution in the whole row, although some singular points may make the distribution curve not completely coincide. Instead of calculating the Kullback-Leibler Divergence between rows, the use of row mean can save a lot of computation and time without wasting the correlation between rows. The row clustering method needs to transmit extra information to the decoder to record the change of row order, facing the same problem, using the mean also needs to transmit the mean information of each line to the decoder. In practice, we will compare the extra coding amount and the actual revenue value brought by the row mean value. When the gain is greater than the original, we will choose to add the line mean information as the context.

Specifically, in the process of building context model, there will be the problem of context dilution, so we need to design a suitable quantization method for mean value so that solve the problem of context dilution, this is a dynamic programming problem and the optimization objective of the quantization of a discretely distributed random variable is to minimize the distortion. The expression for the objective is:

1 $$\begin{aligned} \begin{aligned} Ed(x,Q(x))=\sum \limits _{i}d(x_i,Q(x_i))p(x_i) \end{aligned} \end{aligned}$$

where $x_i$ is the value of *x* that have nonzero probability, $Q(x_i)$ is the quantization value of $x_i$, and $d(\cdot ,\cdot )$ is a specific distortion measure. We can define a condition set $M_{\theta }=\{m_i,i=1...N\}$ to indicate that each specific $m_i$ corresponds to a specific *E* value. Define a quantized set $Q_{\theta }=\{q_k,k=1...K\}$, where *K* represents the quantized variable, so $M_{\theta }=\bigcup \limits _{k=1}^{K}q_k$, $q_i\bigcap {q_j}=\varnothing$, if $i\ne {j}$. Therefore, each subset $q_k$ corresponds to a partition of $m_i$, which has $p(q_k)=\sum _{m_j\in {q_k}}p(m_j)$. The expressions for *H*(*X*|*M*) and *H*(*X*|*Q*) are as follows:

2 $$\begin{aligned}&H(X|M)=\sum _{m_i\in {M_{\theta }}}p(m_i)\sum _{x}p(x|m_i)log_2\frac{1}{p(x|m_i)} \end{aligned}$$

3 $$\begin{aligned}&H(X|Q)=\sum _{q_k\in {Q_{\theta }}}p(q_k)\sum _{x}p(x|q_k)log_2\frac{1}{p(x|q_k)} \end{aligned}$$

it can be seen that the $q_k$ generated from *M* to *Q* contains all the $m_i$, so $p(x|q_k)$ can be regarded as the quantized value of $p(x|m_i)$. Following the Equ.(1), we can get the context quantized as:

4 $$\begin{aligned} L=\sum _{m_i\in {M_{\theta }}}d(p(x|m_i),Q(p(x|m_i)))p(m_i) \end{aligned}$$

where *L* is the quantization objective function, and minimizing *L* means obtaining at least locally optimal quantization results. The optimization objective of the context quantization becomes: for a given number of quantization levels *K* $(K<N)$, find an optimum partition scheme for $m_{i}$, $(i=1,\ldots N)$, then calculate the optimum quantization values $Q(p(x|m_i))$ for all *K* partitions so that the Equ.(4) is minimized.

**Fig. 5:**
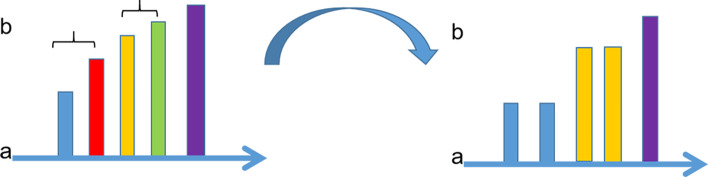
The way of row mean quantization

By calculating the dynamic programming problem, we get a result that is suitable for the test data. In order to improve the computational efficiency in the actual compression process, we use the calculated result as the quantization method. If necessary to improve the compression ratio for the specified file, users can solve the optimization problem separately and get the best way to quantify it. Finally, our method of quantifying row characteristics as shown in Fig.5:

It can be seen that the quantization method in this case is very similar to the lossy compression which joined thresholds. The difference is that we quantify the row characteristics without affecting the lossless decoding, just extract the correlation features between the rows and using the method of dynamic programming to get a better result. The final quantitative method which include the current value *q* is:

5 $$\begin{aligned} &\bullet {if \qquad (q<30)\qquad \qquad \quad then\ q=30}\\&\bullet {else \quad if (30\le q<32)\qquad then\ q=32}\\&\bullet {else \quad if (32\le q<34)\qquad then\ q=34}\\&\bullet {else \quad if (34\le q<36)\qquad then\ q=36}\\&\bullet {else \quad if (36\le q<38)\qquad then\ q=38}\\&\bullet {else \qquad \qquad \qquad \qquad \qquad \quad q=q}\\ \end{aligned}$$

**Fig. 6:**
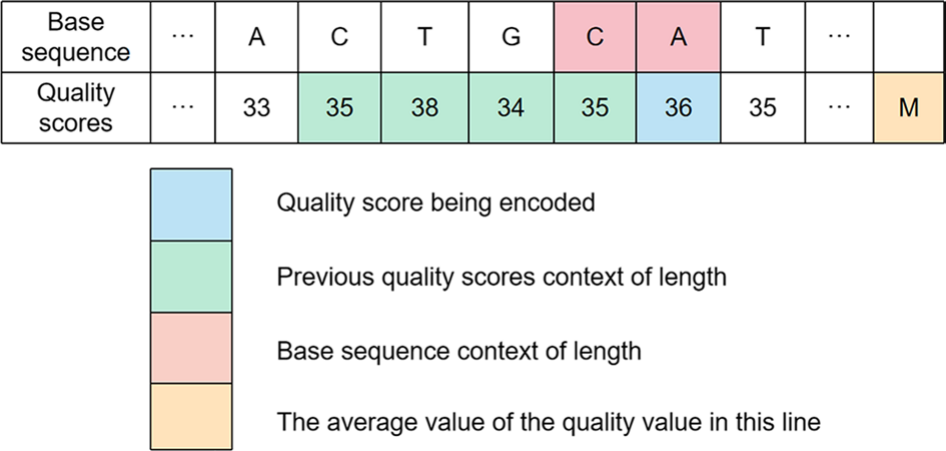
Composite context modeling strategy

On the other hand, the distribution of quality score is random and the waveform will be not smooth transition influenced by quality score values. So different from modeling by the waveform, we can start with sequencing principle for these singular points. [21] reveals that quality score between adjacent bases is usually similar and the probability distribution of the quality score is affected by the base distribution. Considering that there are some natural similarities in the process of obtaining nucleotide sequences, the distribution of bases is regarded as a criterion to measure whether there is a singular point in the quality score, which is used to simulate the stationarity between two symbols. The increase in the base order will cause the model to grow exponentially, and balancing the model size and effect improvement rate, we choose the second-order to describe the correlation between base and quality score. In FASTQ file, a quality score corresponds to a base and the conditional entropy is:

6 $$\begin{aligned}&-log_2\prod ^n_{i=1}p(x_i|j_i,j_{i-1})<-log_2\prod ^n_{i=1}p(x_i|j_i) \end{aligned}$$

where $j_i$ is the base value of the quality score $x_i$ of the current code and this formula shows that the influence of base on entropy. After synthesizing all the context models, we provide the final composite context modeling strategy in Fig.6 and the ACO algorithm in Algorithm 1.

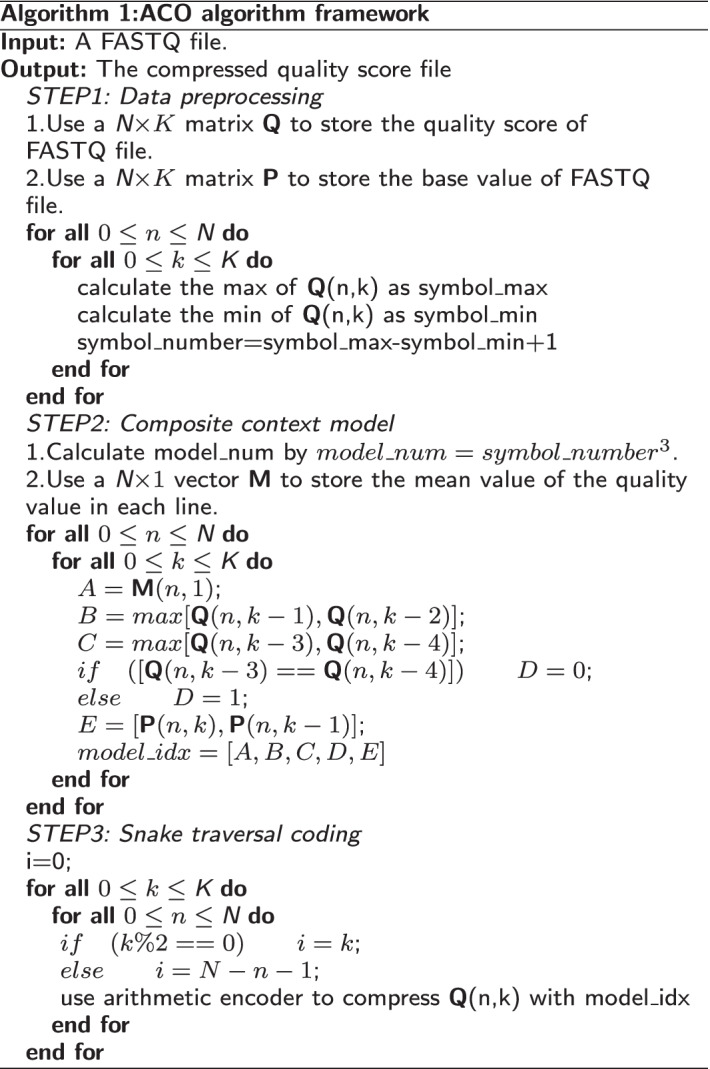



**Table 1:** Descriptions of 6 FASTQ datasets used for evaluation

Run ID	Sequencing platform	FASTQ size(bytes)	Read length	Quality size(bytes)
NA12878_2	BGISEQ-500	134363357648	2*100	56983386200
ERR2438054_1	BGISEQ-500	133406591610	2*150	47097570000
ERR174324_1	Illumina HiSeq 2000	57800970448	2*101	22580690796
ERR174331_1	Illumina HiSeq 2000	57210954538	2*101	22350322320
ERR174327_1	Illumina HiSeq 2000	54724344869	2*101	21379957043
ERR174324_2	Illumina HiSeq 2000	57800970448	2*101	22580690796

**Table 2:** All algorithmic compression results for NGS data sets

Run ID	Ratio	gzip	7z	gtz	quip	fqz-q1	fqz-q2	fqz-q3	Spring	ACO
NA12878_2	CR(%)	48.55	49.66	38.47	38.48	39.08	38.59	38.35	39.68	**36.38**
	BPQ	3.88	3.97	3.08	3.08	3.13	3.09	3.07	3.17	**2.91**
ERR2438054_1	CR(%)	46.23	47.11	37.09	36.52	37.08	36.71	37.63	37.07	**34.55**
	BPQ	3.70	3.77	2.97	2.92	2.97	2.94	2.92	3.01	**2.76**
ERR174324_1	CR(%)	36.58	36.94	25.47	26.14	27.30	25.81	24.90	26.39	**23.86**
	BPQ	2.93	2.96	2.04	2.09	2.18	2.06	1.99	2.11	**1.91**
ERR174331_1	CR(%)	36.55	36.91	25.45	26.11	27.27	25.77	24.86	26.37	**23.87**
	BPQ	2.92	2.95	2.04	2.09	2.18	2.06	1.99	2.11	**1.91**
ERR174327_1	CR(%)	35.53	35.88	24.56	25.31	26.39	24.90	24.02	25.45	**22.97**
	BPQ	2.84	2.87	1.96	2.02	2.11	1.99	1.92	2.04	**1.84**
ERR174324_2	CR(%)	38.47	38.81	27.07	27.52	28.89	27.37	26.35	28.03	**25.52**
	BPQ	3.08	3.10	2.17	2.20	2.31	2.19	2.11	2.24	**2.04**

## Results and discussion

In this section, we have compared the performance of our algorithm ACO with other state of the art algorithms and report the results. We have compared our algorithm with general purpose compression algorithms like gzip and 7zip and also a set of algorithms specific to the domain namely GTZ, fqzcomp and quip. We have restricted our focus to lossless compression, and have not evaluated a number of promising lossy methods, nor methods only capable of compressing nucleotide sequences. For the fqzcomp algorithm, we compare the results of q1, q2 and q3 compression modes and for GTZ algorithm, it does not display the quality score compression results separately, so we compare the normal mode. It is important to note that ACO has more advantages in compressing aligned quality score and does not accept any input other than a raw FASTQ file. At the same time, we do not bring into comparison algorithms that accept any reference genome. The datasets used in our experiments are downloaded in FASTQ format from the National Center for Biotechnology Information - Sequence Read Archive (NCBI-SRA) database [22] and are presented in Table 1.

All the experiments were run on a server with a Inter Core i9-9900K CPU 3.60GHz processor, 32GB of RAM, 2TB disk space and Ubuntu 16.04. All algorithms are compared in terms of compression rate (CR) and bits per quality value (BPQ). The CR and BPQ is defined as follows:

7 $$\begin{aligned}&CR=\frac{L_{after}}{L_{begin}}\times 100\% \end{aligned}$$

8 $$\begin{aligned}&BPQ=8*CR \end{aligned}$$

where $L_{after}$ indicates the compressed file size, $L_{begin}$ indicates the size of the file before compression, compression results of all algorithms on the NGS datasets are summarized in Table 2.

Table 2 gives an improvement of ACO relative to each comparison algorithm, and further reflects the advantages of the method we use by compression ratio. The best results in Table 2 have been bolded. Compared with Gzip, the file size is reduced by an average of 32.01%, and the average file size is reduced by 32.93% compared with the 7-Zip under the optimal setting. The results show that the proposed ACO algorithm achieves better results on six representative data. Particularly, ACO obtains an average compression ratio of 27.67%, resulting in an over 72.33% size reduction in the quality score data. At the same time, the average 2.21 BPQ result is much smaller than the original 8 BPQ in ASCII format. Two evaluation criteria indicate that ACO has achieved the best compression results for the different methods of the same document. According to the differences platforms, the ACO algorithm proposed in this paper sets different modes and processing strategies, which makes the compression efficiency higher.

## Conclusion and future works

This paper introduces ACO, a lossless quality score compression algorithm based on adaptive coding order. ACO traverse the quality score along the most relative directions and use compound context modeling strategy to achieve the state-of-the-art lossless compression performances. However, the current ACO version, especially the proposed compound context modeling strategy is proposed for the second generation sequencing machines. For the third generation sequencing data, the compound context models may be modified to genomes quality score, but the context dilution problem may be appears as the increasing of context models. An alternative solution maybe using the deep learning technique to estimate the marginal probability of every quality score to replace the current context modeling. In our further works, we will concentrate on both of the above two strategies and extend ACO to the third generation sequencing data. In addition, how to choose the best way for different data to automatically calculate the mean value as context information and apply to data containing only 8 quality values will also be our next research work.

## Data Availability

Project name: ACO. Project website: https://github.com/Yoniming/ACO. Operating systems: Linux or Windows. Programming language: C/C++. Other requirements: GCC compiler and the archiving tool ‘tar’. License: The MIT License. Any restrictions to use by non-academics: For commercial use, please contact the authors. All datasets are downloaded from SRA of NCBI. All data supporting the conclusions of this article are included within the article and its additional files.

## Abbreviations

NCBI: National centre for biotechnology information
NGS: Next-generation sequencing
WGS: Whole-genome sequencing
QS: Quality score
CR: Compression ratios
BPQ: Bits per quality value
